# Supplementary material for: Content-rich biological network constructed by mining PubMed abstracts
Source: BMC Bioinformatics. 2004 Oct 8;5:147. doi: 10.1186/1471-2105-5-147 (PMC528731; doi:10.1186/1471-2105-5-147)
Supplement: Additional File 2 — The original results of the above study (non-essential files are deleted to keep the file size under the limit set by BMC bioinformatics). [file 1471-2105-5-147-S2.bz2 › chilibotAdditionalFile2/dip40/388ID11297552E9862/html/PDGFRA_PDGFB.html]

 


 **PDGFRA** and **PDGFB** 
  
Found 113 abstracts in PubMed, retrieved 40.  
 

 What does Google say? 
 PDF only 
| .edu only 

---

**Interactive relationship** (e.g. stimulation, inhibition, etc)

**Non-interactive relationship** (e.g. studied together, co-existance, homology, etc.)

- Interestingly, absence of Nkx2.2 expression leads to a ventral expansion of the Olig1 Olig2 expression in neuroepithelial cells into the Nkx2.2 domain and a consequent increase in the production of Olig1 Olig2 positive and platelet derived growth factor  [ **PDGFB** ]  receptor alpha  [ **PDGFRA** ]  positive oligodendrocyte progenitors.  Ref: 11526078 Development, 2001
- Content and activity of cAMP response element binding protein regulate platelet derived growth factor  [ **PDGFB** ]  receptor alpha  [ **PDGFRA** ]  content in vascular smooth muscles.  Ref: 12130557 Endocrinology, 2002
- Here we show that approximately 35% 14 of 40 of GISTs lacking KIT mutations have intragenic activation mutations in the related receptor tyrosine kinase, platelet derived growth factor  [ **PDGFB** ]  receptor alpha  [ **PDGFRA** ]   **PDGFRA** .  Ref: 12522257 Science, 2003
- In this report, we describe the distribution of the platelet derived growth factor  [ **PDGFB** ]  receptor alpha  [ **PDGFRA** ]  PDGFRalpha by immunolocalization in the embryonic day 10.5 mouse heart and defects in heart development associated with the absence of this receptor in the Patch mouse.  Ref: 11246404 Science, 11246404
- One of the genes was the alpha subunit of the platelet derived growth factor  [ **PDGFB** ]  receptor  **Pdgfra** .  Ref: 12605401 J Neurosci Res, 2003
- We also showed that the A549 lung cancer cells expresses the platelet derived growth factor  [ **PDGFB** ]  receptor alpha  [ **PDGFRA** ] , and the inhibitory effects of Gleevec on A549 cells is likely mediated through inhibition of PDGFR alpha phosphorylation.  Ref: 12537587 Mol Cancer< MedlineTA>Mol Cancer, 2003
- This report identifies a genetic rearrangement in the eosinophilic cell line EOL 1 that results in the expression of a fusion protein comprising an N terminal region encoded by a gene of unknown function with the GenBank accession number NM\_030917 and a C terminal region derived from the intracellular domain of the platelet derived growth factor  [ **PDGFB** ]  receptor alpha  [ **PDGFRA** ]  PDGFRalpha.  Ref: 12808148 Proc Natl Acad Sci U S A, 2003
- Although bone marrow biopsies in these patients showed increased numbers of CD25 mast cells and atypical spindle shaped mast cells, patients with HES and elevated serum tryptase could be distinguished from patients with systemic mastocytosis and eosinophilia by their clinical manifestations, the absence of mast cell aggregates, the lack of a somatic KIT mutation, and the presence of the recently described fusion of the Fip1 like 1 FIP1L1 gene to the platelet derived growth factor  [ **PDGFB** ]  receptor alpha  [ **PDGFRA** ]  gene  **PDGFRA** .  Ref: 12676775 Blood, 2003
- By E11 glial precursors have begun to differentiate and at least two regions of the ventral neural tube containing glial precursor cells can be distinguished, an Nkx2.2 Neurogenin 3 Ngn3 domain and a platelet derived growth factor  [ **PDGFB** ]  receptor alpha  [ **PDGFRA** ]  PDGFRalpha Olig2 Sox10 domain.  Ref: 12237841 Glia, 2002
- We found the PLATELET DERIVED GROWTH FACTOR  [ **PDGFB** ]  RECEPTOR ALPHA  [ **PDGFRA** ] , EARLY GROWTH RESPONSE PROTEIN 1 and INSULIN LIKE GROWTH FACTOR 2 genes as well as several genes associated with MYCC and ERBB2 overexpressed by at least 2 fold in metastatic tumors in both array data sets.  Ref: 12845196 Pediatr Neurosurg, 2003
- Effect of platelet derived growth factor  [ **PDGFB** ]  receptor alpha  [ **PDGFRA** ]  and beta blockade on flow induced neointimal formation in endothelialized baboon vascular grafts.  Ref: 10764412 Circ Res, 2000
- Platelet derived growth factor  [ **PDGFB** ]  receptor A, insulin like growth factor 1 receptor, Janus kinase 1, TYK2, discoidin domain receptor 2, and Lyn were expressed in SF, and the expression of  **PDGFRA** , IGF 1R, and DDR2 in SF of RA were higher than that of osteoarthritis.  Ref: 12419287 J Autoimmun, 2002
- These included absence of expression of CD44, E NCAM, A2B5, epidermal growth factor receptor EGFR, and platelet derived growth factor  [ **PDGFB** ]  receptor alpha  [ **PDGFRA** ]  PDGFR alpha, suggesting that negative selection using cell surface epitopes could be used to isolate stem cell populations from mixed cultures of cells.  Ref: 12435354 Dev Biol, 2002
- We have previously shown that deregulated expression of the platelet derived growth factor  [ **PDGFB** ]  alpha receptor  **PDGFRA**  can be associated with neural tube defects NTDs in both men and mice.  Ref: 12393181 Biochim Biophys Acta, 2002
- Using immunohistochemistry we found that neuroepithelial cells express the platelet derived growth factor  [ **PDGFB** ]  receptor alpha  [ **PDGFRA** ]  PDGFR alpha in the neural plate at embryonic day 8.5 and onwards in the neural tube.  Ref: 11414789 Mol Cell Neurosci, 2001
- Amplification of the platelet derived growth factor  [ **PDGFB** ]  receptor A  **PDGFRA**  gene occurs in oligodendrogliomas with grade IV anaplastic features.  Ref: 10850862 J Neuropathol Exp Neurol, 2000
- By immunocytochemistry platelet derived growth factor  [ **PDGFB** ]  receptor alpha  [ **PDGFRA** ]  PDGF Ralpha was not detected in any of the tissues.  Ref: 11165574 Arch Oral Biol, 2001
- The Fgfr3 cells are interspersed with but distinct from platelet derived growth factor  [ **PDGFB** ]  receptor alpha  [ **PDGFRA** ]   **Pdgfra**  positive oligodendrocyte progenitors.  Ref: 12441294 Development, 2003
- Modulation of transforming growth factor beta to platelet derived growth factor  [ **PDGFB** ]  receptor alpha  [ **PDGFRA** ]  of human osteoblasts.  Ref: 11776032 Chin Med J Engl , 2000
- Characterization of the platelet derived growth factor  [ **PDGFB** ]  receptor alpha  [ **PDGFRA** ]  and c kit genes in the pufferfish Fugu rubripes.  Ref: 12592706 DNA Seq, 2002
- Platelet derived growth factor  [ **PDGFB** ]  receptor alpha  [ **PDGFRA** ]  pdgfr alpha gene in zebrafish embryonic development.  Ref: 12128230 Mech Dev, 2002
- Mouse models have indicated that deregulated expression of the gene encoding the platelet derived growth factor  [ **PDGFB** ]  alpha receptor  **Pdgfra**  causes congenital NTDs refs.  Ref: 11175793 Nat Genet, 2001
- The potential role of p38 mitogen activated protein MAP kinase in platelet derived growth factor  [ **PDGFB** ]  receptor alpha  [ **PDGFRA** ]  PDGF Ralpha gene expression was investigated using cultured rat pulmonary myofibroblasts.  Ref: 10807932 J Biol Chem, 2000
- Notably, platelet derived growth factor  [ **PDGFB** ]  receptor alpha  [ **PDGFRA** ]   **PDGFRA**  and members of the downstream RAS mitogen activated protein kinase MAPK signal transduction pathway are upregulated in M tumors.  Ref: 11544480 Nat Genet, 2001
- Semi quantitativereverse transcription polymerase chain reaction was used to compare the relative abundance of 2 transcripts for housekeeping proteins beta actin and ribosomal protein L30 and 3 transcripts for growth factor ligand or receptors platelet derived growth factor  [ **PDGFB** ]  receptor alpha  [ **PDGFRA** ]  PDGFRalpha, basic fibroblast growth factor bFGF, in mature bovine oocytes of high versus low developmental potential.  Ref: 12760483 Can J Vet Res, 2003
- early oligodendroglial markers were chondroitin sulfate proteoglycan antigen and platelet derived growth factor  [ **PDGFB** ]  receptor alpha  [ **PDGFRA** ] .  Ref: 12271492 J Comp Neurol, 2002
- Clones for the porcine homologues of platelet derived growth factor  [ **PDGFB** ]  receptor alpha  [ **PDGFRA** ]  PDGFRalpha and of insulin like growth factor I receptor IGF IR were found during follicle growth both in oocytes and follicle cells.  Ref: 11470859 Mol Hum Reprod, 2001
- In addition, treatment with RA induced expression of the platelet derived growth factor  [ **PDGFB** ]  receptor alpha  [ **PDGFRA** ] .  Ref: 12808116 Lab Invest, 2003
- We show that OLPs in the embryonic mouse forebrain express platelet derived growth factor  [ **PDGFB** ]  alpha receptors  **PDGFRA** , as they do in more caudal regions.  Ref: 11493571 Development, 2001
- OBJECTIVE To investigate the mechanism and the significance of TGF beta in modulating the expression of Platelet Derived Growth Factor  [ **PDGFB** ]  Receptor alpha  [ **PDGFRA** ]  PDGFR alpha in human osteoblasts.  Ref: 11776032 Chin Med J Engl , 2000
- Platelet derived growth factor  [ **PDGFB** ]  receptor alpha  [ **PDGFRA** ]  in ventricular zone cells and in developing neurons.  Ref: 11414789 Mol Cell Neurosci, 2001
- This Foxf1 phenotype correlates with decreased expression of vascular cell adhesion molecule 1 VCAM 1, alpha 5 integrin, platelet derived growth factor  [ **PDGFB** ]  receptor alpha  [ **PDGFRA** ]  PDGFRalpha and hepatocyte growth factor HGF genes, all of which are critical for cell adhesion, migration, and mesenchymal cell differentiation.  Ref: 11809759 J Biol Chem, 2002
- Here we show that Gli1 can activate platelet derived growth factor  [ **PDGFB** ]  receptor alpha  [ **PDGFRA** ]  PDGFRalpha in C3H10T 1 2 cells.  Ref: 11481486 Proc Natl Acad Sci U S A, 2001
- Because neointimal smooth muscle cells express both platelet derived growth factor  [ **PDGFB** ]  receptor alpha  [ **PDGFRA** ]  and beta PDGFR alpha and beta, we designed this study to test the hypothesis that inhibiting either PDGFR alpha or PDGFR beta with a specific mouse human chimeric antibody will modulate flow induced neointimal formation.  Ref: 10764412 Circ Res, 2000
- Gene expression levels for the platelet derived growth factor  [ **PDGFB** ]  receptor alpha  [ **PDGFRA** ]  PDGFRalpha, plasminogen activator inhibitor 1 PAI 1, and stromal cell derived factor 1A SDF1A are constitutively augmented in RSF compared with NSF.  Ref: 12074593 Biochem Biophys Res Commun, 2002
- Platelet derived growth factor  [ **PDGFB** ]  receptor alpha  [ **PDGFRA** ]  PDGFR alpha is present throughout the mesenchyme of early chick limb buds, while its ligand, PDGF A, is expressed in the surrounding epithelium.  Ref: 10842055 Mech Dev, 2000
- Regulation of interleukin 1beta induced platelet derived growth factor  [ **PDGFB** ]  receptor alpha  [ **PDGFRA** ]  expression in rat pulmonary myofibroblasts by p38 mitogen activated protein kinase.  Ref: 10807932 J Biol Chem, 2000
- Genetic disruption of the platelet derived growth factor  [ **PDGFB** ]  receptor alpha  [ **PDGFRA** ]  PDGFRalpha results in defects in multiple tissues in the mouse, including neural crest derivatives contributing to the frontonasal process and the aortic arch.  Ref: 12490557 Development, 2003
